# Recent Advances in *Stimuli*-Responsive Hydrogel-Based Wound Dressing

**DOI:** 10.3390/gels9060451

**Published:** 2023-05-30

**Authors:** Luigia Serpico, Stefania Dello Iacono, Aniello Cammarano, Luca De Stefano

**Affiliations:** 1 Institute of Applied Sciences and Intelligent Systems (ISASI), National Research Council, Via P. Castellino 111, 80131 Naples, Italy; 2 Materias Srl, Corso N. Protopisani 50, 80146 Naples, Italy; 3 Institute of Polymers, Composites and Biomaterials (IPCB), National Research Council, P.le E. Fermi 1, 80055 Portici, Italy

**Keywords:** hydrogels, wound healing, wound dressing, *stimuli*-responsive, bibliometric analysis

## Abstract

Polymeric materials have found increasing use in biomedical applications in the last decades. Among them, hydrogels represent the chosen class of materials to use in this field, in particular as wound dressings. They are generally non-toxic, biocompatible, and biodegradable, and they can absorb large amounts of exudates. Moreover, hydrogels actively contribute to skin repair promoting fibroblast proliferation and keratinocyte migration, allowing oxygen to permeate, and protecting wounds from microbial invasion. As wound dressing, *stimuli*-responsive systems are particularly advantageous since they can be active only in response to specific environmental *stimuli* (such as pH, light, ROS concentration, temperature, and glucose level). In this review, we briefly resume the human skin’s structure and functions, as well as the wound healing phases; then, we present recent advances in *stimuli*-responsive hydrogels-based wound dressings. Lastly, we provide a bibliometric analysis of knowledge produced in the field.

## 1. Introduction

In the last decade, polymeric materials have found large applications in the biomedical field as delivery systems (DSs) [1,2]. Applied locally as topical DSs, they can provide a transdermal delivery (TD) of active molecules, allowing them to reach systemic circulation while avoiding the first pass-effect linked to other administration routes [3,4,5]. Moreover, they allow a sustained drug release over time and limit adverse/off-target effects. Due to these peculiar characteristics, polymers are particularly suitable for wound dressing [6,7]. In fact, they ensure wound isolation from the external environment, avoid infections, and provide a scaffold for cells involved in skin repair [8,9,10].

Among them, hydrogels are the most used due to their excellent and tunable biochemical and mechanical properties [11,12]. They have: (1) good tissue compatibility, (2) low toxicity, (3) hydrophilic three-dimensional (3D) porous structure that mimics extracellular matrix (ECM), and (4) water-swelling capacity. In addition, hydrogels actively contribute to skin repair: they are able to absorb tissue exudates, promoting fibroblast proliferation and keratinocyte migration, allowing oxygen to permeate, and protecting wounds from microbial invasion [13]. According to their nature, hydrogels can be classified as natural, synthetic, or hybrid [14]. Among the natural ones, chitosan, gelatine, and hyaluronic acid have been largely exploited as wound dressings [15]. They mimic the biological components of ECM and are extremely biocompatible. On the other hand, synthetic hydrophilic polymers—such as poly(ethylene glycol) (PEG), poly(acrylic acid) (PAA), and poly(vinyl alcohol) (PVA)—have been used since they have more controllable chemical and mechanical properties, offering greater reproducibility in fabrication [16].

Some hydrogels are *stimuli*-responsive matrices, allowing a release of embedded molecules triggered by a variation in environmental conditions due to chemical or physical agents. Thus, they have been largely studied and utilized as smart wound dressing for *stimuli*-responsive drug release.

This review first presents the human skin structure and the different phases of wound healing. Then, it focuses on recent advances in the use of hydrogels for *stimuli*-responsive wound dressing. Furthermore, in this work, an overview of knowledge produced in basic and applied science and technology is given through a bibliometric analysis.

## 2. The Skin

**Figure 1:**
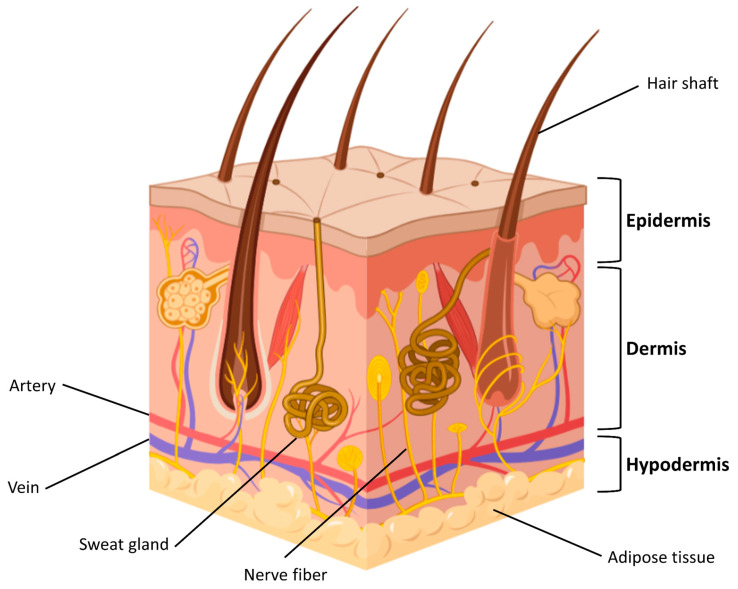
Skin structure (Figure created with Biorender).

The skin is the largest organ of the body and performs different crucial functions [17]. Being continuously exposed to external pathogens, it represents the first body’s barrier, and it is involved in key processes, such as temperature regulation, hydration, vitamin D synthesis, protection from physical and chemical dangerous *stimuli*, and immune response [18]. Skin is constituted by three main layers: the epidermis, the dermis, and the hypodermis (Figure 1). The epidermis is the uppermost layer and exerts sensitive and immunological roles. The dermis is the underlying part; it is connective tissue and supports the skin, contributing to its flexibility [19]. The blood vessels transport nutrients and allow an inflammatory response, recruiting inflammatory cells. Below the dermis, the hypodermis is involved in thermoregulation and nutrition storage.

Given its essential functions, every interruption of the skin has to be considered dangerous and properly treated. Skin wounds, indeed, can potentially cause systemic inflammation and dangerous reactions, such as sepsis [20]. Consequently, the wound healing process—to restore skin integrity—is essential [21].

### Wound Healing Phases

**Figure 2:**
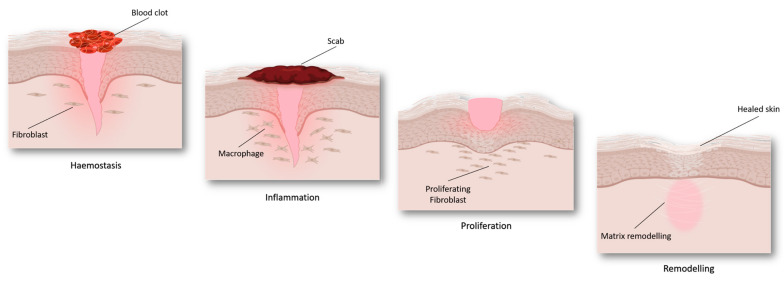
Wound healing steps: haemostasis, inflammation, proliferation, and tissue remodeling (Figure created with Biorender).

Wound healing is a complex and multifactorial process that involves different biological pathways [22], including inflammation and redox regulation [23]. Wound repair can be divided into four different but partially overlapping steps (Figure 2), namely haemostasis, inflammation, proliferation, and tissue remodeling [24,25].

Haemostasis. It typically begins immediately after an injury occurs: in a few minutes, a blood clot is formed in order to stop the bleeding. Platelets are key cells in this phase: they seal off the injured blood vessel—stopping the bleeding—and activate the chemotactic recruiting of inflammatory cells, such as macrophages and leukocytes. Moreover, platelets release cytokines and growth factors that, in turn, provoke the proliferation of smooth muscle cells and fibroblasts, starting the repair of the injured area, and promoting the inflammatory response [26].

Inflammation. Inflammatory cells participate in microorganism killing, favoring successful healing [27]. Furthermore, the recruited and activated immune cells start releasing a series of growth and haemostatic factors that, in turn, stimulate both the blood vessels repairing and the inflammatory response. This environment increases reactive oxygen species (ROS) production and, subsequently, leads to oxidative stress. The inflammatory phase generally follows and overlaps with the proliferative phase.

Proliferation. It is characterized by the macrophage switch from an inflammatory to a proliferative phenotype [28]. Thus, the subsequent production of specific growth factors stimulates the proliferation of involved cellular types, forming a new granulating tissue on the wound surface. Meanwhile, the basal cells migrate to the surface to regenerate new epithelial tissue.

Tissue remodeling. Remodeling and maturation phases lead to the completion of the wound repair. During these phases, the components of the extracellular matrix are restored, and the proliferation is significantly reduced to the normal homeostatic equilibrium [28]. Meanwhile, collagen is deposited on the wound site contributing to normal tissue restoration.

Most wounds end with a complete reformation of the corresponding normal tissue and generally heal in 4–6 weeks. However, a series of factors can affect the restorative process and end up in impaired healing by transforming the lesion into a chronic condition over time. Among them are the presence of vascular, metabolic, and autoimmune diseases, as well as age and ongoing drug therapy. Thus, the use of materials able to enhance wound healing has been shown to be effective in promoting tissue repair and avoiding scar tissue formation.

## 3. Wound Dressing

Wound dressing is a crucial issue in wound management that has a huge impact on health spending. Historically, wound dressing was intended as an inert barrier to prevent wound infection from external agents, and it was represented by gauze or tissue band-aids that did not actively change the wound’s status. For the first time in 1962, George Winter introduced the concept of wet dressing. He observed that covering skin wounds with polythene films accelerated the healing process with respect to air-exposed injuries [29,30]. This evidence showed that keeping the injury site moisturized could enhance tissue repair by promoting cell migration [31]. These results prompted the interest of the scientific community in developing smart wound dressing systems over the years. To date, several materials have been explored and exploited to get dressings that can actively participate in the wound-healing process [32,33]. The new generation of wound dressings includes devices that can sense and respond to the wound environment’s *stimuli* [33]. Recently, *stimuli*-responsive hydrogels have found a large application in the treatment of chronic wounds, such as diabetic ones. The latter still represents a serious challenge: about 20% of diabetics worldwide suffer from poor wound healing [34] with severe associated complications and high costs. 

**Figure 3:**
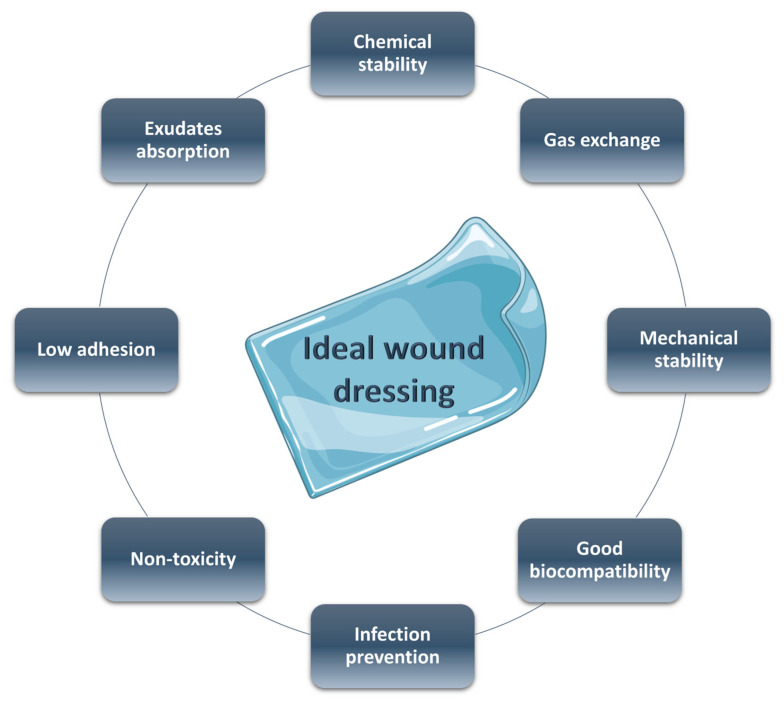
Ideal features of a wound dressing.

Smart materials have been proven to be very effective in improving the repair of these wounds due to their capacities to change mechanical properties, swelling ability, and hydrophilicity in response to different *stimuli*, such as temperature, pH, light, and so on [35]. An ideal wound dressing needs the characteristics reported in Figure 3: (a) good biocompatibility, (b) adequate physical and mechanical properties, (c) surface microstructure in order to permit cell adhesion and differentiation, (d) good moisture adsorption to guarantee the necessary wet environment, (e) low adhesion to the wound tissue to a facile and non-painful removing, (f) non-toxicity, (g) optimal gas exchange between the damaged tissue and the environment, and (h) antibacterial properties [36,37].

Here we illustrate hydrogel-based dressings that meet many of the required points. Hydrogels are defined as crosslinked polymeric 3D networks that are able to absorb a large amount of water or aqueous fluids (such as exudates). They maintain their structure and flexibility, guaranteeing a moisturized environment while enhancing the repairing process [16,38]. Indeed, on the one hand, hydrogel films serve as a barrier, preventing microorganism invasion; on the other hand, they permit gas exchange, avoiding anaerobic bacteria growth [13,21,22,23,26,27,39]. Furthermore, by designing the crosslinking degree—and consequently, the material porosity—it is possible to modulate the release rate of embedded molecules. The water absorbing degree is directly related to characteristic functional groups of the polymers, such as hydroxyl (–OH), carboxylic (–COOH), and amidic (–CONH–) functionalities [40]. Surface chemical functionalization allows modifying the hydrogel permeability in response to specific external *stimuli*, consequently affecting the release kinetics [41]. Taking into account all these considerations, many hydrophilic polymers—such as chitosan, hyaluronic acid, and PEG—have been utilized to form hydrogel-based dressings.

Besides, injectable forms of hydrogels can be easily prepared and used to fill irregular wounds. Moreover, the ones with adhesive properties can promote healing by bonding the wound edges.

## 4. *Stimuli*-Responsive Systems

*Stimuli*-responsive hydrogels are promising smart materials able to change their properties as a response to external variations, such as temperature, pH, light, redox-, enzyme-, magnetic-, and multi-responsive materials [42,43]. Due to this characteristic, they find a large application in the biomedical field and, in particular, as smart wound dressings, being able to improve wound healing (Table 1). They are also exploited in regenerative medicine, cancer treatment [44,45], drug delivery [46,47], sensing [48,49], and more. *Stimuli* can be classified mainly as endogenous and exogenous. The former arises within the organism and includes changes in pH, redox gradient, temperature, and enzyme concentration. Exogenous *stimuli* are artificially applied from outside the body and comprise light, magnetic or electric fields, and so on.

### 4.1. pH-Responsive

The skin’s physical structure, fundamental for the performance of its functions, consists of two main layers: the epidermis—with the stratum corneum—principally constituted by keratinocytes, and the dermis, composed of fibroblasts, collagen, and other extracellular components. Furthermore, various skin appendages, such as eccrine and sebaceous glands, exert specific functions. The former secrete sweat in response to different *stimuli*; the sebaceous glands produce sebum, which lubricates the skin, also maintaining its surface at a pH value of about five [64]. This resulting acidic pH also referred to as acid mantle, represents a key factor in keeping a healthy skin environment since it contributes to barrier homeostasis and integrity and antimicrobial defense [65]. In addition, in this acidic condition, antimicrobial peptides, an important component of innate immunity, are able to work the most [66].

In light of the above, it is clear that pH is crucial in maintaining the correct function of the skin, and it is a strictly regulated factor. pH, indeed, changes in the different layers, increasing from the surface to the deeper ones. Furthermore, it varies in the different phases of wound healing [67,68]. pH alterations can inhibit necessary enzymes, impairing physiological repair. Still, bacterial infection and colonization—typical of chronic wounds—are also affected by topical pH variations. It is different depending on the skin’s status: healthy skin presents a slightly acidic value (5–6), acute wounds have a pH around 7.4, while chronic ones have a more alkaline pH between 7.3 and 10 (partly due to the proliferating bacterial colonies) [69,70,71].

Based on these findings, several pH-responsive materials have been explored for controlled drug release in both acute and chronic wounds with the aim of improving the healing process. Most of these responsive systems are hydrogel-based since they exploit the numerous advantages peculiar to these materials [6,36]. The active molecules can be loaded into the polymeric matrix and then released under specific conditions.

In a very recent study, Bostanci and collaborators presented photocrosslinked hydrogels based on methacrylated forms of pectin and gelatin (PeMA and GelMA, respectively) for pH-dependent release of curcumin, used as antimicrobial agents. In this system, the presence of carboxylic groups on both polymers as well as the pH-dependent solubility of curcumin, resulted in its modulated release, exerting antibacterial activity and allowing a high cell viability [72].

In 2021 Li et al. [73] developed a pH-responsive injectable hydrogel consisting of N-carboxyethyl chitosan, aldehyde hyaluronic acid, and adipic acid dihydrazide for the treatment of diabetic foot ulcers. The release of insulin, encapsulated in the hydrogel dressing, decreased glucose levels, promoting wound healing. The prolonged pH-driven release of insulin is allowed by the presence of acylhydrazone and imine bonds, which undergo rupture in acidic pH environments and significantly affect the hydrogel networks, favoring the drug release.

Lastly, Ahmadian and co-workers presented a multifunctional gelatin-tannic acid (GelTA) hydrogel as a promising candidate for the treatment of chronically infected wounds. They proposed a safe, reproducible one-pot synthesis of a natural hydrogen-bonded hydrogel with high biocompatibility and pH-dependent release of embedded antioxidants. In addition, the hydrogel showed excellent therapeutic effects on wound repair in vivo, promoting cell migration and proliferation, as well as collagen production, being suitable for wound healing applications [74].

Furthermore, our group recently developed a biocompatible, polymeric thin film for a pH-dependent release of synthetic antioxidant selenium-containing glycoconjugates, with a potential application for wound healing acceleration [75]. The antioxidant activity of the two prepared compounds is related to their peculiar structure, characterized by two active sites: a selenium atom—inserted in a sugar ring—and a phenolic moiety (namely a caffeic acid and a resveratrol residue). Moreover, by embedding these molecules in a biocompatible, blended hydrogel, it was possible to obtain a pH-responsive system. In particular, the caffeic acid-containing compound is released at acute wounds’ pH (7.4), while the resveratrol-containing conjugate is released at pH 9.6, typical of chronic wounds.

### 4.2. ROS-Responsive

ROS are crucial regulating factors of the healing process [76], improving physiological skin repair. ROS, indeed, participate in oxidative bacterial killing promoting angiogenesis and re-epithelialization at the wound site. In addition, ROS acts as secondary messengers inducing the activation of several transcription factors, such as nuclear factor kappa B (NF-kB), which is one of the main actors of inflammation. Nevertheless, excessive levels of ROS cause oxidative damage, impairing the correct healing; this phenomenon is especially observed in chronic wounds where a prolonged state of oxidative stress occurs [77]. Targeting oxidative stress in chronic wounds by restoring a redox equilibrium has been proven effective in improving proper wound repair [78]. Taking this evidence into account, antioxidant approaches have been explored to evaluate the related effects in healing acceleration. In this part, we summarize recent studies in which the design of ROS-responsive wound dressings has been largely investigated and used for this purpose.

The well-known property of selenium (Se) to undergo a hydrophobic-hydrophilic transition under oxidation is exploited in Se-containing block copolymers [79]. When oxidized, indeed, Se is converted into more polar selenoxides and/or selenones. Moreover, compared to other chalcogens, Se-based materials are particularly advantageous due to the lower bond energy of C–Se (244 kJmol^−1^) and Se–Se (172 kJmol^−1^). In addition, the Se-Se bond is susceptible both to reducing and oxidizing conditions: it is cleaved and oxidized to seleninic acid in the presence of oxidants and reduced to selenol in a reducing environment. This responsive disassembling of Se-containing block copolymers makes them suitable biomaterials for controlled drug release.

Ma et al. developed ROS-responsive selenium-containing block copolymer for drug delivery applications. They prepared a diselenide containing polyurethane (PUSeSe) block copolymer finally terminated by PEG (PEG-PUSeSe-PEG) incorporating fluorescent Rhodamine B as a drug model. The PEG-PUSeSe-PEG showed to be disassembled—releasing their cargo—in a mild oxidative environment (0.1% *v*/*v* H_2_O_2_) [80]. Moreover, the material was also responsive to reductant *stimuli* (GSH 0.01 mg/mL). This characteristic confers key amphiphilic properties that are useful in wound healing acceleration.

ROS-responsive biomaterials that are able to scavenge ROS present a real potential in diabetic wound treatment. Diabetic wounds are a chronic health issue that leads to hindered skin regeneration.

Zhu et al. prepared an injectable, amphiphilic block copolymer, including poly(ethylene glycol)-b-poly(propylene sulfide) (PEG-PPS), able to self-assemble in aqueous solution becoming a stable hydrogel [81]. This star-PEG-PPS scaffold fills the entire wound bed, providing structural support for cellular infiltration and tissue regeneration. In their system, oxidized polypropylene sulfide acts as a reactive oxygen species (ROS) quencher in reactive wounds, favoring the repair.

Zhao and collaborators have proposed a poly (vinyl alcohol) (PVA)-based, ROS-responsive hydrogel to promote healing in diabetic wounds [82]. Due to the insertion of a ROS-responsive linker, namely N1-(4-boronobenzyl)-N3-(4-boronophenyl)-N1, N1, N3, N3-tetramethylpropane-1, 3-diaminium (TPA); they obtained a ROS-responsive degradation of the final system, with the consequent controlled release of the embedded active molecules. The obtained hydrogel showed an effective ROS-scavenging activity by decreasing ROS levels.

In a recent study, Hu et al. [83] proposed a ROS-responsive hydrogel-based nanocomposite with antibacterial properties to use in infected wounds. They prepared a polymer constituted of polyacrylic acid (PA) containing AgNPs and Fe^2+^/Fe^3+^-contained polyglutamic acid (PG). The resulting hydrogel, named PAAg-PGFe, showed a semi-solid status in physiological conditions, while the exposure to H_2_O_2_ caused hydrogel breakage with consequent exerting of antibacterial activity. Furthermore, it exhibited no toxicity in mammalian cells in vitro, whereas it demonstrated quick wound healing and pathogen removal in vivo experiments.

In a very interesting study, carboxymethyl chitosan (CMCTS)-based hydrogel crosslinked by a ROS-sensitive linker (thioketone group, Tk) and loaded with curcumin (Cur) Cur@CMCTS-Tk was explored as an effective wound dressing. This system exhibited topical and controlled release of Cur, a potent antioxidant, removing ROS in the wound site and showing a potential application in burn wound treatment both in vitro and in vivo [84].

**Figure 4:**
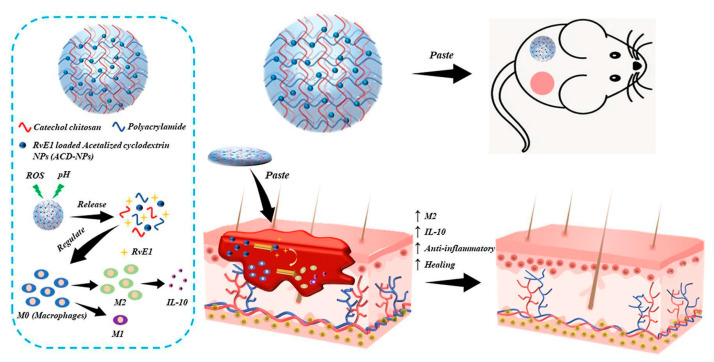
Double responsive hydrogel network. Reproduced from open access ref. [85].

Alternately, double-network (DN) hydrogels as a double-responsive drug delivery system for chronic wound treatment were proposed [85]. The DN system was obtained by incorporating polyacrylamide (PAM) into catechol chitosan (C-CS) hydrogel, crosslinked, using Bis(acryloyl)cystamine (BAC) and Cystamine (Cys), respectively. PAM enhanced the system’s mechanical properties, while Cys disulfide bonds made the hydrogel network ROS-responsive. Moreover, catechol groups enhanced tissue and cell adhesion, promoting wound healing. In addition, embedded pH-responsive nanoparticles—prepared by acetalized cyclodextrin—were used for the pH-dependent release of anti-inflammatory molecules (Figure 4).

### 4.3. Light-Sensitive

Recently, *stimuli*-responsive hydrogels have emerged as promising smart wound dressings. Photo-responsiveness is particularly advantageous since the *stimulus* of biomaterials is constituted by light. Light is a non-invasive form of energy that does not require chemical agents to exert its effect. It can be finely modulated depending on exposure time and intensity, as well as on the selected wavelength [86]. Thus, the combination of light energy and hydrogel responsiveness led to the development of several efficient systems for wound treatment. A recent and comprehensive review of photoactive hydrogels for wound healing improvement has been published by Maleki et al. [87]. Here, we focus our attention on some examples of antibacterial and light-responsive hydrogels for chronic wound treatment.

Shi and collaborators just presented a new dual light-responsive cellulose nanofibril (CNF)-based in situ hydrogel wound dressing (CNF-DLRIHWD). In the first place, CNFs were grafted with antibacterial agents polyaminopropyl biguanide and Protoporphyrin IX (PAPB and PpIX, respectively). CNFs were then integrated with Prussian blue nanoparticles (PBNPs), Pluronic^®^ F127 (F127), and hydroxypropyl methylcellulose (HPMC). This system contributes to wound healing by exerting an excellent antimicrobial effect, joined with photodynamic and photothermal therapies. Moreover, the resulting nanocomposite was shown to be able to counteract bacterial biofilms, promoting the repair of infected chronic wounds [88].

Likewise, Yang et al. [89] developed a dual-functional system with antibacterial and wound-healing dressing features. A dodecyl-modified chitosan hydrogel, linked to dialdehyde-functionalized PEG via a Schiff base reaction, was added with WS2 nanosheets—a photothermal agent—and loaded with ciprofloxacin—an antimicrobial drug. Under the irradiation of near-infrared (NIR) light, WS2 generates heat, providing a photothermal treatment and photo-response drug therapy simultaneously. The antibiotic, released in a spatially and temporally controlled mode, leads to bacterial death and eliminates the inflammatory response, showing a good anti-oxidation activity that, in turn, promotes wound healing.

**Figure 5:**
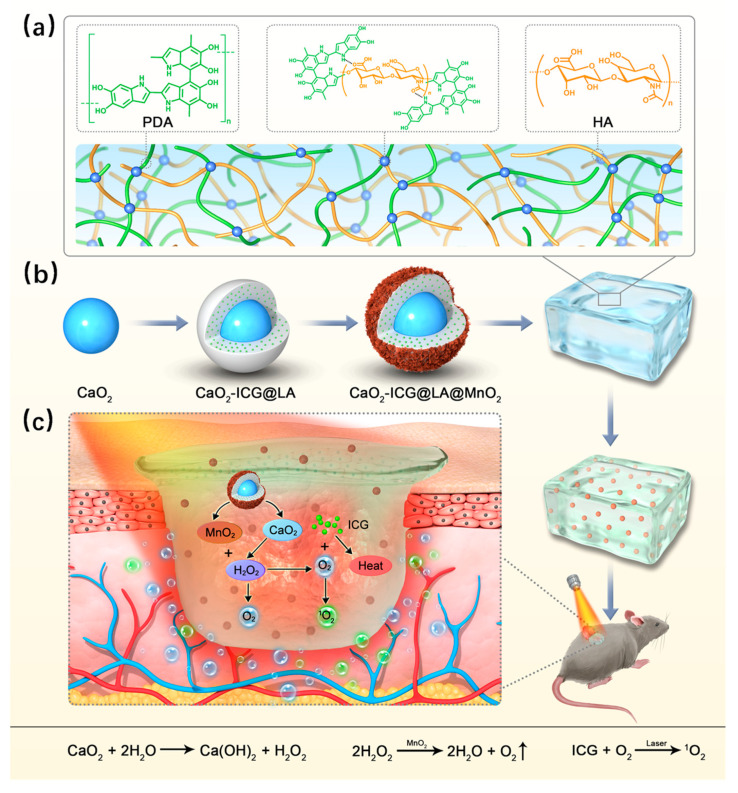
Preparation and application of the NIR-responsive nanocomposite. (**a**) Synthesis of the hydrogel (PDA-HA). (**b**) Assembling of CaO_2_ based nanocomposite. (**c**) Nanocomposite hydrogel as wound dressing. Reproduced from open access ref. [90].

Recently, Li et al. [90] proposed a NIR-responsive system consisting of polydopamine-hyaluronic acid (PDA-HA) hydrogel-loaded calcium peroxide-indocyanine green added to lauric acid and manganese dioxide (CaO_2_-ICG@LA@MnO_2_) nanoparticles. In detail, a core shell, in which CaO_2_ is partially linked with ICG, is covered by LA and then combined with MnO_2_. By irradiating the system with NIR laser light, an on/off release of O_2_ is obtained. In detail, the induced photothermal effect causes the LA to melt at 40 °C. Consequently, CaO_2_, now in an aqueous environment, is hydrolyzed to H_2_O_2_, which in turn produces O_2_. In this system, LA hydrophobicity restrains the O_2_ release, while MnO_2_ is the reaction catalyst and MnO_2_ is the reaction catalyst (Figure 5).

### 4.4. Glucose-Responsive

Glucose-responsive hydrogels find a specific application in diabetic chronic wound treatment. In diabetics, skin repair is often impaired due to elevated blood glucose levels [91,92]. Hyperglycemia, indeed, induces chronic inflammation, altered angiogenesis, and augmented production of glycation end products and strong oxidative stress conditions [93]. All these co-existent factors contribute to a compromised wound healing process characterized by prolonged inflammation and reduced re-epithelization [94,95,96]. Diabetic wounds, indeed, still represent a health care challenge: the diabetic foot ulcer causes disability, amputation, and ultimately death if it is not properly treated [97]. Due to this, several hydrogel-based wound dressings have been proposed for improved repair [98].

Xu and collaborators proposed a novel glucose-responsive hydrogel with antioxidant properties to enhance wound repair in these wounds [60]. They grafted gallic acid (GA) on the surface of chitosan (CS) chains and then embedded it in a PEGDA hydrogel matrix. Moreover, they modified polyethyleneimine (PEI) NPS with glucose-sensitive phenylboronic acid (PBA) and loaded the resulting NPs with insulin. The NPs were linked to the CS-GA chains through the borate bond between the GA hydroxyl groups and phenylboronic acid groups. The resulting platform was able to release insulin in hyperglycemic conditions. In addition, the antioxidant activity, due to the embedded polyphenol, improved the repair by re-establishing a redox balance.

**Figure 6:**
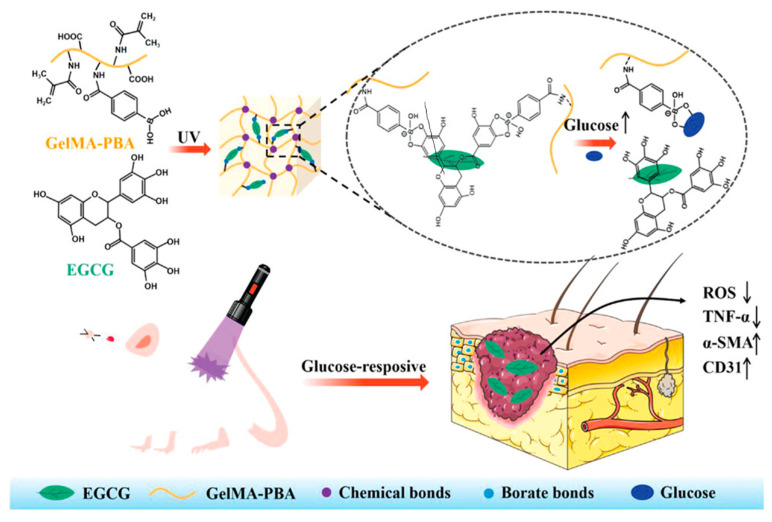
Preparation of the glucose-responsive hydrogel. Reproduced from open access ref. [99].

Similarly, Chen et al. recently presented a device for the controlled release of (−)-epigallocatechin-3-gallate (EGCG), a natural polyphenol with proven antioxidant activity, in the presence of high glucose levels (Figure 6). The system was prepared by modifying Gelatin methacryloyl (GelMA) with 4-carboxyphenyboronic acid (CPBA). Then, the inclusion of EGCG allowed the formation of glucose-responsive boronic ester bonds between the PBA groups of the matrix and the ortho-dihydroxy groups of EGCG. Due to this peculiar interaction, the resulting material showed an EGCG sustained release in a glucose-responsive manner, accelerating wound healing by eliminating excessive ROS in the wound site [99]. In vitro and in vivo studies confirmed that they had a high grade of biocompatibility and no toxicity paving the way to a possible clinical application.

Yang and collaborators proposed a GOx-containing, multifunctional metalorganic drug-loaded hydrogel (DG@Gel). When applied to a diabetic wound, the GOx causes a decrease in glucose levels in the wound microenvironment by converting glucose into gluconic acid and hydrogen peroxide. This reaction provokes a decrease in pH levels that—in turn—leads to the material’s swelling with a consequent release of loaded active molecules. Due to this, the designed system presented a synergistic effect in diabetic wound healing acceleration, both in vitro and in vivo experiments [100].

### 4.5. Thermosensitive

According to the classification of Zamboni et al. [101], thermo-responses are part of the second-generation hydrogels and show numerous advantages in controlled release systems. They are usually crosslinked by non-covalent interactions, and their physical state is temperature driven. This can induce the sol–gel transition, allowing the perfect adaptability of the gel on the wound site, even for the injectable type avoiding surgical implantation. Incorporating the drug in a flow state guarantees a homogeneous dispersion; meanwhile, the rapid gel formation—via sol–gel transition, often occurring at physiological temperature—prevents an initial burst release, providing a sustaining delivery. Thermo-responsive hydrogels can swell/shrink according to the environmental temperature [102], with related changes in volume due to the hydrophobic/hydrophilic functional groups present in the gels’ chemical structure.

Among synthetic temperature-sensitive hydrogels, the ones based on poly (N-isopropylacrylamide) (PNIPAAM) are the most extensively studied [103]. They have a lower critical solution temperature (LCST), close to the body temperature (32 °C). Above this value, the hydrogen bonds break, and the solution turns into a gel-like state due to hydrophobic interactions [104]. A hybrid hydrogel, obtained by a combination of a short peptide (I3K) with PNIPAM [105], resulted in a system—I3K self-assembled fibrils entangling with PNIPAM—forming a 3D network. An antibacterial peptide G(IIKK)3I-NH2, loaded as a model drug, assessed the sustained and linear release in the aqueous environment at higher temperatures in the wound site. Indeed, positive controlled thermo-responsive hydrogels (*i.e.,* PNIPAM) allow a more rapid drug release at increased temperature—characteristic of the chronic wound’s inflammatory state—and slower delivery at a lower temperature.

Analogously, natural hydrogel chitosan (CTS)-based systems have been deeply investigated, especially due to their excellent biocompatibility. In particular, the presence of β-glycerolphosphate (β-GP) is reported to allow the sol–gel transformation in CTS-based hydrogel at physiological temperature (37 °C) [106]. In a recent study [107], a β-GP-CTS-based thermo-responsive polymer led to a significant decrease in bacterial population in infected wounds, in vivo experiments with extensively drug-resistant (XDR). Bacteria were clinically isolated from burn patients, accomplished with an acceleration of wound healing, re-epithelialization, and wound closure.

However, the temperature guide, while conferring the dynamic behavior, makes these hydrogels potentially unstable, as they could turn into the liquid phase at temperatures lower than the LCST or higher than the upper critical solution ones (UCST), failing the sustained release. Therefore, although they are widely applied as thermo-responsive polymers, their use can be affected by rapid dissolution in aqueous solutions, which strongly limits a prolonged release.

To overcome this limit, in a recent work, Yan and collaborators developed a hydrogel composed of poly(Nisopropylacrylamide166- co-n-butyl acrylate9)-poly(ethylene glycol)-poly(N-isopropylacrylamide166-co-n-butyl acrylate9) copolymer and silver-nanoparticles-decorated reduced graphene oxide nanosheets [108]. The inorganic/polymeric dual network confers to the system stability with respect to the sol–gel transition, even at temperatures lower than LCST. The stable wound dressing activity is detected in the healing of a methicillin-resistant Staphylococcus aureus-infected skin lesion.

## 5. Bibliometric Data Collection and Analysis

Bibliometric analysis is a research field that has grown exponentially in recent years and, to date, represents a valid instrument for the analysis of large amounts of data.

In this review, we provide a general overview of the research on the development of hydrogels as wound dressings over the last decades, using bibliometric methods. We used Elsevier’s Scopus, known for its vast collection of peer-reviewed literature spanning various scientific domains, as a database. The bibliometric analysis includes co-occurrence and network analysis to summarize the progress in the field and define the emerging trends and contributions of authors, journals, institutes, or countries using specific data samples. The software VOSviewer (www.vosviewer.com, accessed on 27 March 2023) was used to develop a visual map of the co-occurrence of keywords and researchers.

**Figure 7:**
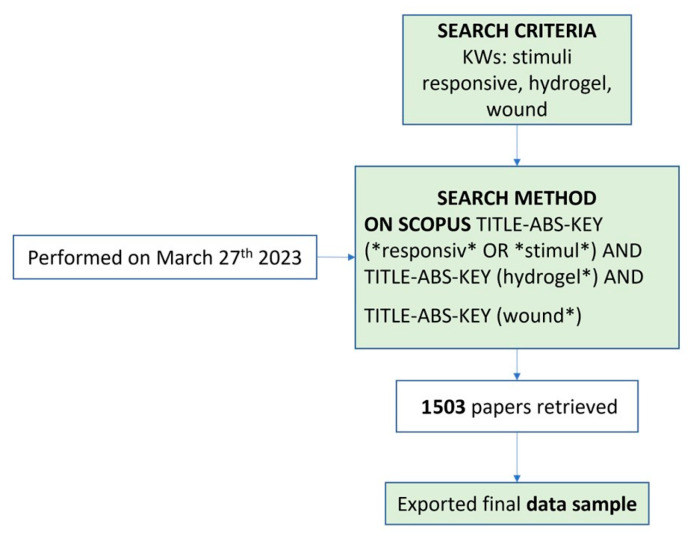
Data sample collection strategy.

In this cross-sectional study, the publication data were collected on a single day (27 March 2023) and downloaded as a csv file from Scopus. The proposed method utilizes programming scripts to automatically extract bibliographic records from scientific publications through the open-access Scopus Database API Interface. The gathered information was then organized using Excel^®^ programming functions, and the data sample collection strategy is shown in Figure 7. The search terms were determined by the query TITLE-ABS-KEY (*responsiv* OR *stimul*) AND TITLE-ABS-KEY (hydrogel*) AND TITLE-ABS-KEY (wound*).

**Figure 8:**
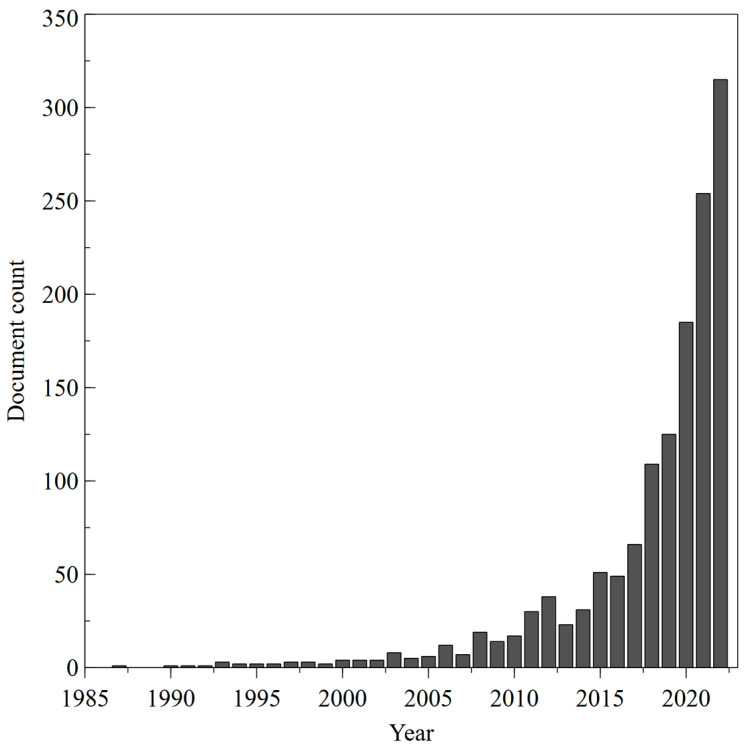
Annual global publication in transdermal drug delivery.

A total of 1503 articles related to *stimuli*-responsive hydrogel-based wound dressing were retrieved from Scopus. The recorded data were also processed by using bibliometric visualization software to extract and analyze publication outputs and create knowledge maps. In the current study VOSviewer version 1.6.19 (www.vosviewer.com, accessed on 27 March 2023) was used to show bibliographic information on researchers, research institutions, countries, citations, and keywords extracted from Scopus csv files by creating network maps. Moreover, co-authorship and co-occurrence analysis were performed to identify the main topics and investigate hotspots in the literature. In detail, co-authorship study reveals patterns of collaboration among countries, while co-occurrence of keywords analysis—using the frequency of multiple words—identifies how close they are, thereby pointing out hot topics and trends in the specific discipline [109]. In this study, we analyzed the top high-frequency keywords in the retrieved publications to explore the research hotspots in transdermal drug delivery. The output of annual publications on this topic is shown in Figure 8.

The annual global publications increased from one in 1987 to 315 in 2022, with an annual growth rate of 30% in 2020, also due to the global pandemic emergency. COVID-19, in fact, generated a global health emergency, and the scientific community has reallocated research programs to find innovative solutions to prevent and fight COVID-19. The collected data support the hypothesis that the COVID-19 crisis induced a sudden increase in research output in the biomedical research areas, including *stimuli*-responsive hydrogel-based wound dressing [110].

On the other hand, the rising number of publications in the first decade of the 2000s can be ascribed to the approval of new TDD active molecules by the Food and Drug Administration (FDA) [111]. The approval process of new drugs requires extensive research and testing to ensure their safety and efficacy. This often generates new scientific knowledge that, in turn, can be shared through scientific papers. Furthermore, when the FDA authorizes a new drug, it can spark interest and additional research in related fields, resulting in a further increase in the number of scientific publications.

**Figure 9:**
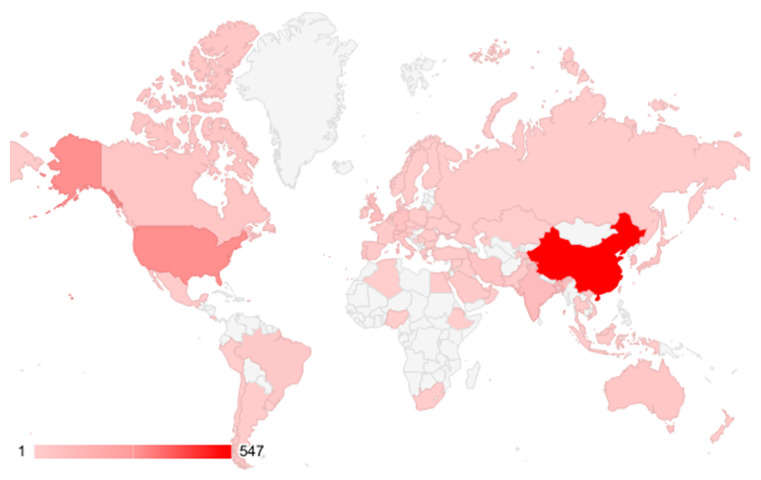
Top countries in publishing works about hydrogel-based wound dressing.

Regarding the absolute number of publications, researchers in China (547), the United States (293), and India (95) produced the most works. Figure 9 shows the top 70 countries where the most papers on *stimuli*-responsive hydrogel-based wound dressing were published.

In Table 2, the top 10 funding sponsors, such as government agencies or non-profit organizations, are reported. China and the United States result as the major contributors to scientific dissemination in this field due to the large public funds invested.

As shown in Table 3, the highest number of papers are published in material science disciplines. In the study on wound dressing systems, researchers with specific expertise can develop hydrogels that absorb wound exudate, provide a moist environment for wound healing, and release therapeutic agents to promote healing [20]. Material scientists can also incorporate different *stimuli*-responsive mechanisms into hydrogels to make them sensitive to specific environmental conditions, develop new biocompatible materials that promote wound healing and prevent infection and use nanotechnology to create hydrogels with unique properties, such as improved mechanical strength and controlled drug release.

**Figure 10:**
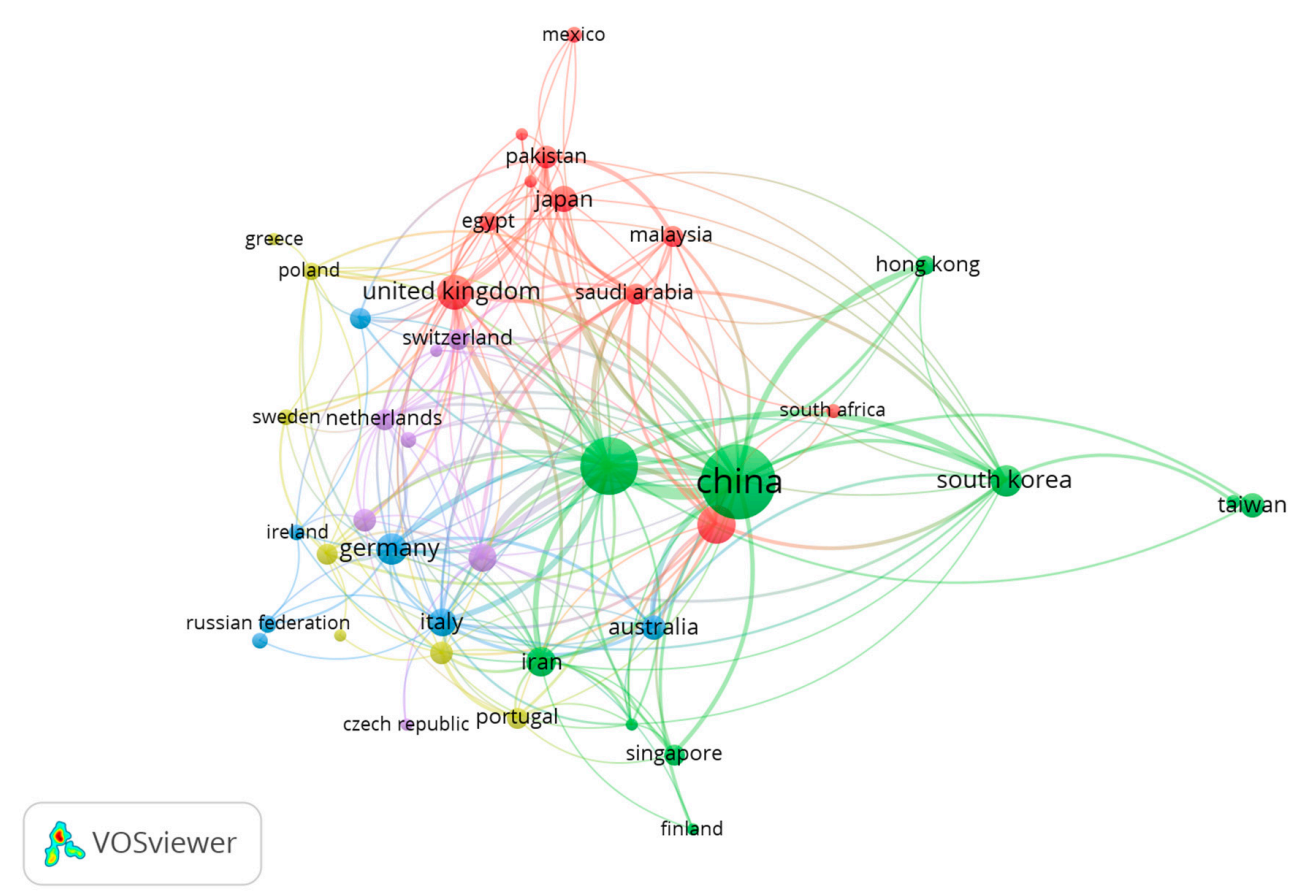
The co-authorship network of Countries.

The links of international collaborations on *stimuli*-responsive hydrogel-based wound dressing research were visualized by VOSviewer—to clearly show the co-authorship analysis among the countries (Figure 10). In the network visualization, countries are represented by circles, whose size is correlated to the country’s importance: the higher the weight of a country, the larger the circle. Minor countries are not displayed to avoid overlapping. The circle’s color is defined by the cluster to which the country belongs. Lines between countries represent links: the 1000 strongest links are reported. In this graphical representation, the distance between two countries reveals approximately their relatedness in co-authorship links: the closer to each other two countries are, the stronger their relationship is.

In detail, the network, including 42 countries, displayed five clusters identified with different colors and connected to each other through co-authorship links. The largest cluster (green)—consisting of 10 countries, 1121 articles and 52,392 citations—is centered over China and the United States. India and the United Kingdom are part of the second largest cluster (red), consisting of 10 countries and 335 articles. It is noteworthy that the USA exhibited the most significant number of cooperating partners (30).

The co-occurrences indicate the number of documents in which a keyword occurs. Keywords in research papers are words that define the research topic and are used to make more discoverable the scientific article. In this study, VOSviewer extracted and clustered the top 50 keywords, as shown in Table 4.

**Figure 11:**
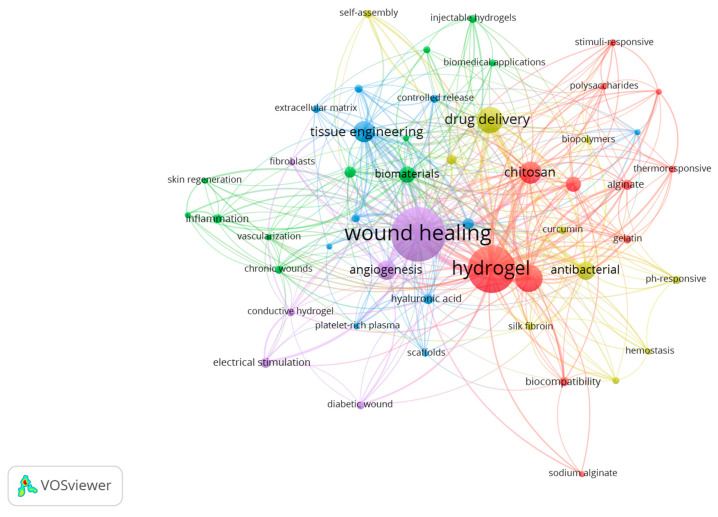
The co-occurrence cluster analysis of the top keywords.

In VOSviewer visualization maps (Figure 11), each node is represented by a labeled circle. A maximum of 1000 lines was set to display the 1000 most robust links [112].

The keywords wound healing (306), hydrogel (246), drug delivery (93), tissue engineering (66), and biomaterials (43) were placed at the center of the network.

Cluster 1—in red—represents scientific papers in which wound dressing solutions with *stimuli*-responsive abilities have been obtained by using chitosan and alginate, biopolymers that are often the building blocks for the hydrogel matrix [113]. Some studies investigate the self-healing and thermo-responsive properties of these hydrogels adaptable to the changing environment of the wound site, promote healing and prevent infections [114].

Cluster 2—in green—mainly represents research in *stimuli*-responsive hydrogels for wound healing, and it is focused on the use of biomaterials to promote tissue regeneration and reduce inflammation in chronic wounds. By designing hydrogels—able to respond to specific cues in the wound microenvironment (e.g., the release of therapeutic agents)—researchers aim to create more effective wound-healing treatments that can address the underlying causes of chronic wounds [115].

Cluster 3—in blue—symbolizes the papers in which the main issues are tissue engineering, extracellular matrix, and growth factors in the context of *stimuli*-responsive hydrogels for wound healing. *Stimuli*-responsive hydrogels have been studied for their potential applications in tissue engineering and wound healing due to their ability to mimic the ECM and provide a favorable environment for cell growth and proliferation. Collagen and hyaluronic acid are two key components of the ECM, and they are commonly used as building blocks for hydrogels in tissue engineering. Hydrogels made of these materials have been shown to promote cell adhesion and proliferation and can be modified to exhibit controlled release of key growth factors [116], such as transforming growth factor-beta (TGF-beta), platelet-derived growth factor (PDGF), and vascular endothelial growth factor (VEGF).

Cluster 4—in yellow—represents studies on *stimuli*-responsive hydrogels for antibacterial purposes since they can release antimicrobial agents to prevent infection [117]. For example, some drugs (e.g., curcumin) can be incorporated into hydrogels and released in response to changes in pH (pH-responsive hydrogels), which indeed occur in a wound environment.

Cluster 5—in purple—symbolizes research in which *stimuli*-responsive hydrogels have been studied for the treatment of diabetic wounds by promoting angiogenesis and accelerating the healing process also by using electrical stimulation. That is a promising approach to enhance the therapeutic effects of these materials, improving their efficacy in wound healing applications [63].

## 6. Conclusions

The recent increase in material science research prompted the development of new systems for smart wound dressing. Polymers, in particular hydrogels, have been proven to be suitable materials for this application since they have several advantageous characteristics. Hydrogels are able to guarantee a properly moisturized environment while promoting skin repair. Moreover, they can form *stimuli*-responsive matrices, allowing a release of embedded molecules in response to specific environmental *stimuli*. Thus, they have been largely studied and utilized as smart wound dressing for *stimuli*-responsive drug release. In this review, we summarized the recent advances in the field with a focus on the newest smart wound dressings.

It emerges that pH-responsive materials are particularly suitable for the treatment of both acute and chronic wounds, which are characterized by peculiar pH levels. These systems exploit the intrinsic properties of the materials to develop clinically suitable devices.

ROS-responsive biomaterials are very promising in wound healing applications, especially in chronic diabetic wounds, where the microenvironment is characterized by a persistent oxidative stress condition. Considering their widespread feasibility, these systems could have a huge positive impact on patients’ lifestyles, as well as on health spending.

Light-responsive matrices have been proven to be efficient therapeutic systems in wound healing by using an environmentally friendly and highly modulable source.

Glucose-responsive smart hydrogels are particularly useful in diabetic wound repair since they are able to release active molecules in hyperglycaemic conditions. This has been proven to be significantly effective in improving the repair of impaired wounds in diabetics, with potential positive spillovers on health costs and patients’ wellness.

Temperature-sensitive hydrogels showed their efficacy in inflamed sites. They swell/shrink according to the temperature, and related water content affects the crosslinking—hydrophobic interactions, hydrogen bonds and the mesh size [118], making them suitable as drug delivery systems in wound dressing with excellent spatial and time control.

Lastly, the bibliometric analysis performed showed an increased interest in the research field over the last decades. This evidence is reported in co-authorship and the co-occurrence maps, as well as in all the related collected data.

## Figures and Tables

**Table 1:** *Stimuli*-responsive systems.

*Stimulus*	Hydrogel-Based System	System Operation	System Performance	Ref.
Temperature/ NIR	poly(N-isopropyl acrylamide)- polydopamine NPs	phase transitions and volume changes in response to NIR	improved cell affinity, good tissue adhesiveness, and growth factor/protein immobilization ability	[50]
Temperature/ pH	hydroxypropyl chitin/tannic acid/ferric ion (HPCH/TA/Fe)	pH-dependent thermosensitivity due to the coordination between TA and Fe^3+^	inhibited bacterial infection	[51]
pH	oxidized dextran-dopamine (OD-DA)+AgNPs+deferoxamine (DFO)	pH-responsive Schiff base structure	antimicrobial capacities to gram-positive and gram-negative bacteria and improved angiogenesis	[52]
pH	carboxylated agarose/tannic acid hydrogel cross + zinc ions	intermolecular ionic and H-bonding strongly affected by pH value	antibacterial and anti-inflammatory properties and low cytotoxicity	[53]
pH	dialdehyde carboxymethyl cellulose (DCMC)/Tobramycin (TB)/borneol/mono-6-(2-hydroxy-3-(trimethylammonio)propyl)-β-cyclodextrin (BN/EPTAC-β-CD)	Imine bonds break in response to the weakly acidic environment	anti-inflammatory function	[54]
Temperature	human collagen-peptide (RHC)/chitosan	thermoreversible sol–gel transition	promoted cell infiltration, vessel formation, and wound healing in second-degree burns	[55]
NIR/ Temperature	MXene nanofibers (MNFs)/dopamine-hyaluronic acid hydrogel (H)/vascular endothelial growth factor (V)/diallyl trisulfide as H_2_S donor (DA)	V release from the MXene nanofibrous skeleton induced by NIR light exposure and photothermal effect	inhibition of excessive neovascularization and extracellular matrix deposition at the wound site	[56]
Temperature	sodium alginate (SA)/gelatin (GT), protocatechualdehyde/ferric ions	temperature-dependent dynamic hydrogel crosslinked through Schiff base bond, catechol-Fe coordinate bond, and strong interactions between GT and SA	shape adaptability, antibacterial activity, and good biocompatibility facilitated post-wound-closure care	[57]
NIR	polyvinyl alcohol (PVA)/poly prodrug (GS-Linker-MPEG)/ up-conversion nanoparticles (UCNP)/ gentamicin sulfate (GS)	NIR light-triggered GS release *via* cleavage of physical UV-susceptible crosslinks between PVA and GS-Linker-MPEG; thanks to UCNP that converts NIR light to UV light	biocompatibility and antibacterial activity	[58]
Glucose	polyethylene glycol diacrylates (PEG-DA), phenylboronic acid (PBA) modified hyaluronic acid (HA), myricetin (MY)	glucose-triggered release of strongly antioxidant MY and immobilized in dynamic borate bond polyphenol group	efficient ROS-scavenging, ameliorated inflammatory response, accelerated angiogenesis, and increased tissue remodeling	[59]
Glucose	gallic acid (GA) grafted onto chitosan (CS) poly (ethylene glycol) diacrylate (PEG-DA) + phenylboronic acid (PBA), modified polyethyleneimine (PEI), insulin NPs	glucose-responsive insulin release through dynamic borate bond between the phenylboronic acid groups on the PEI-PBA and the polyphenol groups on the CS-GA	biocompatibility, antioxidant properties, protection of cells from oxidative damage, promoted angiogenesis, and accelerated wound closure	[60]
Glucose	hyaluronic acid methacrylate (HAMA) with phenylboronic acid (PBA)/catechin (HMPC)	glucose-responsive catechin release allowed by sensitive borate ester bond between HAMA-PBA and catechin	biocompatibility, antioxidant capability, elimination of intracellular reactive oxygen species, cell protection from oxidative stress damage, angiogenesis promotion, and reduced inflammatory responses	[61]
ROS	PBA grafted sodium alginate (Alg-PBA)/polyvinyl alcohol (PVA)/sodium hyaluronate PBA (HA-PBA)	ROS-responsive drug release due to network structure destruction under H_2_O_2_ and diffusion of Doxycycline hydrochloride	antibacterial activity and improvement of infected wounds’ treatment	[62]
pH/ROS	caffeic acid-grafted ε-polylysine (CE) + phenylboronic acid-grafted oxidized dextran (POD)	hydrogel network breakage due to hydrolysis of ROS-sensitive Schiff base and boronic ester bonds under acidic and oxidative conditions	inhibition of inflammatory response and promotion of wound healing in infected diabetic wounds	[63]

**Table 2:** Top 10 public funding sponsors.

Funding Sponsor	Country	Documents
National Natural Science Foundation of China	China	368
Fundamental Research Funds for the Central Universities	China	94
National Key Research and Development Program of China	China	82
National Institutes of Health	United States	73
China Postdoctoral Science Foundation	China	49
National Science Foundation	United States	42
National Research Foundation of Korea	Korea	30
National Heart, Lung, and Blood Institute	United States	25
Natural Science Foundation of Shaanxi Province	China	21
National Institute of Arthritis and Musculoskeletal and Skin Diseases	United States	20

**Table 3:** Top 10 subject areas.

Subject Area	Documents	%
Materials Science	765	24
Engineering	544	17
Biochemistry	421	13
Chemistry	351	11
Chemical Engineering	325	10
Medicine	257	8
Pharmacology, Toxicology and Pharmaceutics	205	6
Physics	142	4
Environmental Science	36	1
Immunology and Microbiology	31	1

**Table 4:** Clusters of the top keywords.

Keywords				
Cluster 1	Cluster 2	Cluster 3	Cluster 4	Cluster 5
alginate biocompatibility chitosan gelatin hydrogel injectable polysaccharides self-healing sodium alginate *stimuli*-responsive thermoresponsive wound dressing	3d printing biomaterials biomedical applications chronic wounds inflammation injectable hydrogels macrophages nanotechnology skin regeneration tissue regeneration vascularization	biodegradable collagen controlled release extracellular matrix growth factors hyaluronic acid infection platelet-rich plasma regenerative medicine scaffolds tissue engineering	antibacterial antimicrobial biopolymers curcumin drug delivery hemostasis nanoparticles pH-responsive self-assembly silk fibroin	angiogenesis conductive hydrogel diabetic wound electrical stimulation fibroblasts wound healing

## Data Availability

The raw/processed data required to reproduce these findings cannot be shared at this time as the data also forms part of an ongoing study.
